# Supplementary material for: Improving the accuracy of single-trial fMRI response estimates using GLMsingle
Source: eLife. 2022 Nov 29;11:e77599. doi: 10.7554/eLife.77599 (PMC9708069; doi:10.7554/eLife.77599)
Supplement: Transparent reporting form [file elife-77599-transrepform1.docx]

***eLife’s* transparent reporting form**

We encourage authors to provide detailed information *within their submission* to facilitate the interpretation and replication of experiments. Authors can upload supporting documentation to indicate the use of appropriate reporting guidelines for health-related research (see [EQUATOR Network](http://www.equator-network.org/%20)), life science research (see the [BioSharing Information Resource](https://biosharing.org/)), or the [ARRIVE guidelines](http://www.plosbiology.org/article/info:doi/10.1371/journal.pbio.1000412) for reporting work involving animal research. Where applicable, authors should refer to any relevant reporting standards documents in this form.

If you have any questions, please consult our Journal Policies and/or contact us: [editorial@elifesciences.org](mailto:editorial@elifesciences.org).

**Sample-size estimation**

- You should state whether an appropriate sample size was computed when the study was being designed
- You should state the statistical method of sample size computation and any required assumptions
- If no explicit power analysis was used, you should describe how you decided what sample (replicate) size (number) to use

Please outline where this information can be found within the submission (e.g., sections or figure legends), or explain why this information doesn’t apply to your submission:

No new data were collected from human subjects for this manuscript; only existing datasets were analyzed. The sample size for our analyses is dictated by the sample size of the NSD and BOLD5000 datasets (see pages 16-17). The goal of the present work is to demonstrate improvements in data quality afforded by the proposed analysis method. We show strong evidence that the method improves data quality consistently across voxels, brain areas, subjects, and datasets.

**Replicates**

- You should report how often each experiment was performed
- You should include a definition of biological versus technical replication
- The data obtained should be provided and sufficient information should be provided to indicate the number of independent biological and/or technical replicates
- If you encountered any outliers, you should describe how these were handled
- Criteria for exclusion/inclusion of data should be clearly stated
- High-throughput sequence data should be uploaded before submission, with a private link for reviewers provided (these are available from both GEO and ArrayExpress)

Please outline where this information can be found within the submission (e.g., sections or figure legends), or explain why this information doesn’t apply to your submission:

Results in this paper (data quality improvements) were replicated in 8 unique subjects from 2 different datasets. No data were excluded from analyses performed in this paper, with the following exceptions: (1) For computational convenience, we used the first 10 (of up to 40) scan sessions from each NSD subject; since the 40 scan sessions have essentially the same experimental design, this should not induce any bias. (2) One subject (subject CSI4 from BOLD5000) was excluded from the decoding analysis in Fig 6 due to having an insufficient number of stimulus repetitions available.

**Statistical reporting**

- Statistical analysis methods should be described and justified
- Raw data should be presented in figures whenever informative to do so (typically when N per group is less than 10)
- For each experiment, you should identify the statistical tests used, exact values of N, definitions of center, methods of multiple test correction, and dispersion and precision measures (e.g., mean, median, SD, SEM, confidence intervals; and, for the major substantive results, a measure of effect size (e.g., Pearson's r, Cohen's d)
- Report exact p-values wherever possible alongside the summary statistics and 95% confidence intervals. These should be reported for all key questions and not only when the p-value is less than 0.05.

Please outline where this information can be found within the submission (e.g., sections or figure legends), or explain why this information doesn’t apply to your submission:

All statistical analyses and quantifications are described in detail in the manuscript (see Methods: pages 18-21), and extensive plots showing results for individual voxels, areas, and subjects are provided. We quantify effects using standard measures (e.g. mean, SEM, correlation). All main findings (e.g. improved reliability due to GLMsingle) are demonstrated in individual subjects, and the plots provide sufficient detail to understand the variability in the precise sizes of these effects.

(For large datasets, or papers with a very large number of statistical tests, you may upload a single table file with tests, Ns, etc., with reference to sections in the manuscript.)

**Group allocation**

- Indicate how samples were allocated into experimental groups (in the case of clinical studies, please specify allocation to treatment method); if randomization was used, please also state if restricted randomization was applied
- Indicate if masking was used during group allocation, data collection and/or data analysis

Please outline where this information can be found within the submission (e.g., sections or figure legends), or explain why this information doesn’t apply to your submission:

Not applicable; the methods we describe in the paper are intended to be applied equally to all subjects.

**Additional data files (“source data”)**

- We encourage you to upload relevant additional data files, such as numerical data that are represented as a graph in a figure, or as a summary table
- Where provided, these should be in the most useful format, and they can be uploaded as “Source data” files linked to a main figure or table
- Include model definition files including the full list of parameters used
- Include code used for data analysis (e.g., R, MatLab)
- Avoid stating that data files are “available upon request”

Please indicate the figures or tables for which source data files have been provided:

The experimental data used in this paper are all previously published and available online at <https://naturalscenesdataset.org> and <https://bold5000-dataset.github.io/website>. The GLMsingle toolbox is available on GitHub at <https://github.com/cvnlab/GLMsingle>. Code used for the analyses demonstrated in this paper is publicly available on GitHub at <https://github.com/jacob-prince/GLMsingle_paper>. Source data files linked to the results plotted in Figures 2-6 are available in an OSF repository at <https://osf.io/d4p98>.
